# *Kingella kingae* and Viral Infections

**DOI:** 10.3390/microorganisms10020230

**Published:** 2022-01-21

**Authors:** Romain Basmaci, Philippe Bidet, Stéphane Bonacorsi

**Affiliations:** 1 Service de Pédiatrie-Urgences, Hôpital Louis-Mourier, APHP, F-92700 Colombes, France; 2 Université de Paris, Inserm, IAME, UMR1137, F-75006 Paris, France; philippe.bidet@aphp.fr (P.B.); stephane.bonacorsi@aphp.fr (S.B.); 3 Microbiologie, Hôpital Robert Debré, APHP, F-75019 Paris, France

**Keywords:** *Kingella kingae*, virus, human rhinovirus, hand foot and mouth disease, children, viral infection

## Abstract

*Kingella kingae* (*K. kingae*) is an oropharyngeal commensal agent of toddlers and the primary cause of osteoarticular infections in 6–23-month-old children. Knowing that the oropharynx of young children is the reservoir and the portal of entry of *K. kingae*, these results suggested that a viral infection may promote *K. kingae* infection. In this narrative review, we report the current knowledge of the concomitance between *K. kingae* and viral infections. This hypothesis was first suggested because some authors described that symptoms of viral infections were frequently concomitant with *K. kingae* infection. Second, specific viral syndromes, such as hand, foot and mouth disease or stomatitis, have been described in children experiencing a *K. kingae* infection. Moreover, some clusters of *K. kingae* infection occurring in daycare centers were preceded by viral outbreaks. Third, the major viruses identified in patients during *K. kingae* infection were human rhinovirus or coxsackievirus, which both belong to the Picornaviridae family and are known to facilitate bacterial infections. Finally, a temporal association was observed between human rhinovirus circulation and *K. kingae* infection. Although highly probable, the role of viral infection in the *K. kingae* pathophysiology remains unclear and is based on case description or temporal association. Molecular studies are needed.

## 1. Introduction

*Kingella kingae* (*K. kingae*), a species of Gram-negative bacteria facultative β-hemolytic coccobacillus, is an oropharyngeal commensal agent of toddlers and the primary cause of osteoarticular infections (OAI) in children in several countries, especially in the 6–23-month age group [1,2,3,4,5].

Several virulence factors and clones have been suggested to be involved in the pathophysiology of *K. kingae* invasive infections [6,7,8,9], but the role of viral infections has also been suggested. An accumulation of data sustain this hypothesis: (i) symptoms of viral infections are frequently concomitant with *K. kingae* infection [1]; (ii) some respiratory viruses have been identified in children experiencing a *K. kingae* infection [10]; (iii) some day-care center outbreaks of *K. kingae* infection followed outbreaks of viral infections [11]; and (iv) a temporal association has been observed between human rhinovirus circulation in the population and the epidemiology of *K. kingae* infection [12].

In this narrative review, we attempt to describe the current knowledge of the interaction between *Kingella kingae* and viral infections.

## 2. Methods

A narrative review based on the published literature was performed. A literature search with free terms related to viral infections, with no quotations (virus; “OR” viral infection; “OR” viral symptoms; “OR” enterovirus; “OR” coxsackievirus; “OR” rhinovirus; “OR” varicella; “OR” influenza; “OR” adenovirus; “OR” parainfluenzae; “OR” metapneumovirus; “OR” Ebstein Barr virus; “OR” cytomegalovirus; “OR” herpes simplex; “OR” parvovirus) combined with “AND” *K. kingae* was conducted using the PubMed database with the PubMed Advanced Search Builder until 9 November 2021. No language restriction was used. Potential articles were screened step-by-step starting with the title, then the abstract, and finally the full text when necessary.

However, this search strategy did not accurately identify those papers on *K. kingae* infections suggesting that the oropharynx was the portal of entry for these infections, and that viral symptoms were frequently concomitant with *K. kingae* infections. To complete the narrative review, these papers were identified from the references linked to the articles found with the search strategy or to recent published review papers on *K. kingae* [1,3,11].

## 3. Literature Review

Overall, only 50 published articles were identified by the search strategy. Thirty-one articles were excluded because they were outside of the relevant topics, 3 were excluded because they specifically described *K. kingae* infection in adult HIV patients, which is very rare, and is not representative of the majority of *K. kingae* infections. Finally, 16 published articles were found to describe the association between *K. kingae* and viral infections. Five additional papers were included in the review.

### 3.1. Viral Symptoms Precede or Are Concomitant with K. kingae Infections

Many authors have described the temporal relationship between viral symptoms and series of *K. kingae* sporadic infection. However, most of these studies do not specify the virus or the specific viral syndrome involved.

As early as 1985, Claesson et al. described that 12/33 (36%) patients with *K. kingae* infection had a history of recent or current upper respiratory tract infection [13]. In addition, two patients had an extremely poor dental status, and data were missing for the 19 remaining cases. Overall, the authors estimated that an obvious possibility of invasion through a damaged mucosal barrier in the oral or nasopharyngeal tract was observed in at least 42% of patients [13].

Similarly, in 1993, Yagupsky et al. observed that 14/25 (56%) patients with *K. kingae* infection had a concomitant upper respiratory tract infection or stomatitis [14]. In addition to seasonal distribution of *K. kingae* infection, the putative role of respiratory virus to facilitate tissue invasion by bacteria was suggested, as previously described with *Haemophilus infuenzae* b [15].

These data were confirmed in 2010, when Dubnov-Raz et al. published a large series of 322 *K. kingae* infections in Israel [16]. In 200 out of 322 (62.1%) episodes, patients had an acute illness reported in the week before or coincidental with the invasive *K. kingae* infection. These were upper respiratory tract infection, pharyngitis, acute otitis media, aphthous stomatitis, vomiting, or diarrhea [16].

In a study assessing oropharyngeal viral carriage in children with a *K. kingae* osteoarticular infection in a 1-year period, Basmaci et al. observed that 10/17 (58.9%) patients presented at least one symptom of upper respiratory tract infection, and that among the seven patients with no symptoms, six carried a respiratory virus [10].

In a small series of *K. kingae* tenosynovitis from three tertiary care centers in France, Switzerland and Israel published in 2018, authors observed that eight out of nine children had viral infection in the preceding month [17].

Overall, more than 60% of reported patients in these studies presented viral symptoms before or concomitantly with a *K. kingae* infection (Table 1) [10,13,14,15,16,17].

Similar results were observed under the specific conditions of *K. kingae* infection occurring in day-care centers. In a review published in 2016, which summarized the data of eight published outbreaks on 27 patients, authors found that previous or concomitant oral ulceration was detected in 10 out of 18 patients (55.6%), for whom the data were available [11].

Knowing that the oropharynx of young children is the reservoir and the portal of entry of *K. kingae*, these results suggested that a viral infection may promote *K. kingae* infection [1,18].

### 3.2. Specific Viral Syndrome or Virus Identification Concomitantly with K. kingae Infections

To go further, some case reports or series describe the association of *K. kingae* with a specific viral syndrome or identify some viruses. However, to the best of our knowledge, only one article has performed a systematic screening of respiratory viruses in patients during *K. kingae* infection, using a FilmArray^®^ respiratory panel [10]. In this paper, the authors described that among 21 *K. kingae* osteoarticular infections in a 1-year period, 19 (90.5%) carried at least one respiratory virus in their oropharynx. The distribution was as follows: human rhinovirus, 12 (57.1%); coronavirus OC43, 4 (19.0%); parainfluenzae (1, 2, 3 or 4), 3 (14.3%); enterovirus, 2 (9.5%); and adenovirus, 2 (9.5%) [10].

Other published articles have focused on one virus or specific viral syndrome. However, very few papers have focused on this topic, and fewer than 50 patients have been fully described. For the sake of clarity and consistency, we chose to describe the literature for each virus independently. The full description of identified viruses or viral syndromes is shown in Table 2.

#### 3.2.1. Human Rhinovirus (HRV)

Three published articles describe the presence of human rhinovirus during *K. kingae* infection, and one assesses the temporal association between these two pathogens (see Section 3.3 below).

The largest series was described above, when HRV was identified in 12/21 children with *K. kingae* OAI between January and December 2013 in France [10]. In addition, the same authors published two atypical cases of *K. kingae* infection in children, with one case involving a soft tissue abscess and one case a femoral Brodie abscess; both patients had concomitant human rhinovirus infection [19]. In 1991, Carden et al. published a case of an 11-month-old girl with metastatic endophthalmitis due to *K. kingae*, and a rhinovirus was isolated from her nasopharynx [20].

#### 3.2.2. Coxsackievirus/Hand–Foot–Mouth Disease (HFMD)

Five papers describe the concomitance of *K. kingae* infection with hand, foot and mouth disease (HFMD) [21,22,23,24] and one paper identifies enterovirus infection [10]. HFMD is a childhood disease caused by human enteroviruses that particularly affects toddlers in the age range of 6–23 months [33]. From April to October 2013, seven children ranging from 10 to 23 months old were diagnosed with *K. kingae* osteoarticular infections in Marseille, France, and with HFMD or stomatitis in the previous weeks. A coxsackievirus-A6 was identified in the stools of one child having HFMD [21]. The same authors described an outbreak of *K. kingae* infections in five toddlers attending the same classroom in a day-care center in Marseille [22]. These patients were probably the same as those in the former study. In this *K. kingae* outbreak, the authors observed that an outbreak of herpangina and HMFD, with an attack rate of clinical infections of 38.3% (8/21), began in the index classroom 2 weeks before the first *K. kingae* case was diagnosed. Moreover, upper respiratory tract infections and fever affected a large number of the index classmates during the same period [22].

In 2007, an outbreak of *K. kingae* infection was observed in a daycare center in the USA [25]. An 11-month-old young girl presented with altered mental status and fever. She was diagnosed with *K. kingae* endocarditis and meningitis, and she had presented with HFMD 3 weeks earlier.

In 2017, an article described the case of a 14-month-old girl with an 11-day history of fever and a 1-day period of vomiting due to a *K. kingae* endocarditis [23]. She was attending a childcare center, where a severe outbreak of HFMD (five toddlers out of 15 attendees) had occurred a few weeks preceding the child’s admission to hospital. The case reported experienced HFMD infection during the outbreak. Of these five children, a 14-month-old boy was also suspected of having *K. kingae* infection.

As previously mentioned, an enterovirus was identified in two patients of the series published by Basmaci et al. [10].

Finally, a case was reported in a 31-year-old woman with *K. kingae* septicemia in the context of oral lesions from coxsackievirus infection, proven by serology [24].

#### 3.2.3. Herpes Simplex Virus (HSV)/Stomatitis

In a study published in 1998, 16 patients with *K. kingae* infection had erosions in the buccal, gingival and/or glossal surfaces [26]. Duration of the stomatitis before blood sampling was 2 to 6 days. In four children with gingivostomatitis, herpes simplex virus was isolated from oral lesions, and in an additional four patients, the clinical data suggested herpetic gingivostomatitis.

A recent paper reported the case of a previously healthy 18-month-old young girl with a diagnosis compatible with herpetic gingivostomatitis and *K. kingae* occult bacteremia [27].

#### 3.2.4. Varicella Zoster Virus (VZV)/Chickenpox

In 1997, Waghorn et al. reported a case of fatal *K. kingae* endocarditis following acute chickenpox with erythrobastopenia and thombocytopenia in a 9-month-old infant. He was first admitted 10 days after developing chickenpox, was discharged after 5 days with initial clinical improvement, but he was readmitted 5 days after discharge with sudden breathing difficulties and cardiorespiratory arrest. No viral test was performed to isolate VZV [28].

In 1998, among patients with stomatitis, Amir et al. identified one *K. kingae* infection in a child concomitantly with a clinically diagnosed VZV infection [26].

More recently, Kampouroglou et al. reported a subacute osteomyelitis by *K. kingae* in a 5-year-old boy after chickenpox infection [29].

#### 3.2.5. Other Viruses and Viral Infections

Other viral infections were more rarely identified concomitantly with *K. kingae* infection. As previously discussed, in the study published by Basmaci et al., different viruses were identified: coronavirus OC43 in 4 (19.0%) children; parainfluenzae (1, 2, 3 or 4) in 3 (14.3%) children; and adenovirus in 2 (9.5%) children [10]. Chosidow et al. reported the case of a 16-month-old boy with recurrent *K. kingae* septic arthritis with a concomitant viral infection with parainflenzae 3 virus [30].

To the best of our knowledge, only one case was associated with Epstein-Barr virus (EBV) and one with influenza virus.

A healthy 2-year-old child presented febrile torticollis with painful 1-cm cervical lymph nodes [31]. Blood tests showed an acute EBV infection. A CT scan associated with MRI showed an inflammatory process at the C1–C2 vertebrae and *K. kingae*-specific PCR was positive on biopsy.

A 14-month-old-girl was admitted with influenza A infection. She was suspected of encephalomyelitis based on fever, prostration, hypertonia, hyperalgesia, urinary retention, and meningitis. She was treated with 5 methylprednisolone bolus. After the last bolus, a systolic cardiac murmur was heard and a *K. kingae* endocarditis was diagnosed, complicated with several cerebral and renal complications [32].

### 3.3. Temporal Association between K. kingae Infection and Circulation of Human Rhinovirus

**Figure 1:**
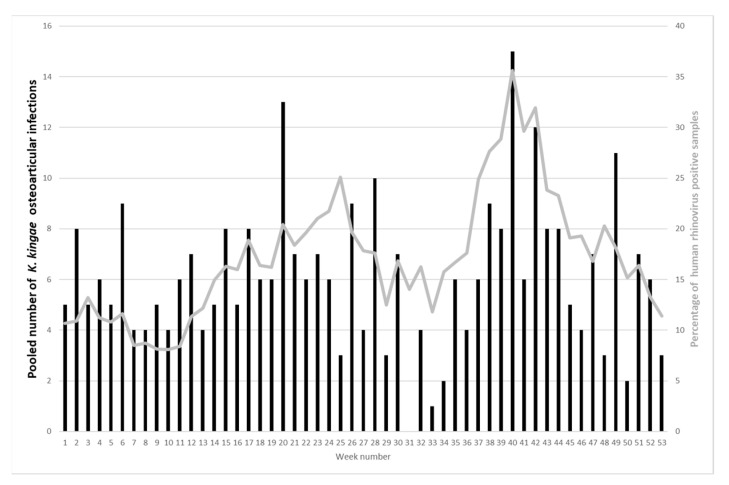
Distribution of the pooled number of *K. kingae* osteoarticular infections (bars) compared with the pooled percentage of human rhinovirus positive samples (line) in Ile-de-France between October 2009 and September 2016.

Recently, a French study analyzed the seasonality of *K. kingae* OAI in two tertiary-care centers in Paris on a 7-year period, including 322 cases of *K kingae* osteoarticular infection and compared the seasonality with the data of respiratory virus detection from the Réseau National des Laboratoires network in coordination with the National Influenza Center of France [12]. The authors observed high activity for both *K. kingae* osteoarticular infection and HRV during the fall (98 (30.4%) and 2401 (39.1%) cases, respectively) and low activity during summer (59 (18.3%) and 681 (11.1%) cases, respectively). Weekly distributions of *K. kingae* osteoarticular infection and rhinovirus activity were significantly correlated (*r* = 0.30; *p* = 0.03), while no significant correlation was found between the weekly distribution of *K. kingae* osteoarticular infection and respiratory syncytial virus, influenza virus, and metapneumovirus [12] (Figure 1).

## 4. Discussion

The peak of incidence of many respiratory viral infections coincides with the age of *K. kingae* carriage and invasive infections [1]. Knowing that the reservoir of *K. kingae* is the oropharynx of young children [1,34], and that *K. kingae* possesses some virulence factors such as type IV pili (allowing adhesion to respiratory epithelium) [6,35,36], and RTX toxin (having a cytotoxic activity able to breach the respiratory epithelium) [7,37,38], it seems plausible that damage to the mucosal layer caused by a viral disease facilitates the entry of *K. kingae* organisms in the bloodstream [1].

In this review, we observed that the majority of available and published data identified human rhinovirus or enterovirus, especially coxsackievirus responsible of HFMD during *K. kingae* infection [10,12,19,20,21,22,23,24], whereas other viruses were less commonly identified.

Of interest, rhinovirus and enterovirus are both members of the Picornaviridae family, which includes nine genera, six of which are pathogenic for humans: enterovirus, rhinovirus, hepatovirus, parechovirus, cardiovirus, and kobuvirus [39].

Three different species of HRV were described: HRV-A, HRV-B, and HRV-C, which was discovered more recently [39,40].

It has previously been described that HRV infection may predispose to bacterial infections. Indeed, temporal correlation between HRV infection and *Streptococcus pneumoniae* has been described [41]. Moreover, HRV infection induces overexpression of platelet-activating factor (PAF) receptor and activation of NF-kB increasing *S. pneumoniae* adherence to the airway epithelial cells [41]. It has also been described that HRV may induce *Staphylococcus aureus* infections [42]. Finally, HRV is able to increase paracellular permeability of airway epithelial cells after infection by disrupting airway epithelial barrier function [43].

Apart from one study that observed a temporal association between HRV and *K. kingae* infections and small series or case reports, no data are available from cellular or animal models to explain the interaction between HRV, respiratory epithelial cells, and *K. kingae*, although further studies would be interesting to address this hypothesis. Moreover, whether an HRV species is more involved in the *K. kingae* pathogenesis remains to be determined.

Enterovirus is a large genus including different species (enterovirus, poliovirus, coxsackievirus) and more than 300 serotypes. Coxsackievirus are organized in two groups: 23 types of coxsakievirus A and six types of coksackievirus B. Coxsakievirus A is responsible for HFMD, whereas coxsakievirus B is often responsible for more severe disease, such as myocarditis.

The effect on coxsakieviruses B on adherence and invasion on Hep-2 epithelial cells was different depending on the bacteria, whereby adherence and invasion increased for *Campylobacter* and *Salmonella* Typhimurium, while they decreased for *Escherichia coli* and *Shigella* [44,45,46]. These scarce data cannot be extrapolated to different serotypes of enterovirus or coxsakievirus nor in respiratory epithelial cells and to the putative interaction with *K. kingae*. To date, only the clinical descriptions that we have reported in this review support the hypothesis of such interaction between both pathogens [10,21,22,23,24,25]; however, further studies are needed to better understand the potential interaction between some serotypes of enterovirus and *K. kingae*.

Finally, our narrative review has some limitations. Despite an increasing available literature on *K. kingae* infection over three decades, very few papers have focused on the concomitance between viral infections and *K. kingae* infections in terms of either epidemiology, microbiology, or molecular studies. A limited number of papers was analyzed in this review, which may not be representative of the majority of *K. kingae* infections and is not sufficient to draw strong conclusions.

Although we observed that more than 60% of reported patients presented viral symptoms before or concomitantly with a *K. kingae* infection, we cannot exclude that this number is overestimated, because this review focused on published papers describing virus and *K. kingae* coinfection.

## 5. Conclusions

Current knowledge suggests that respiratory viral infections play a probable major role in the pathophysiology of *K. kingae* in breaching the respiratory epithelium; however, this hypothesis is based on only a small number of cases or series and temporal association. Molecular studies on cellular cultures would be of great interest in better understanding such mechanisms.

## Figures and Tables

**Table 1:** Number of patients with sporadic *Kingella kingae* infection presenting viral infection or damaged mucosal barrier in published articles.

First Author, Year	Patients with Viral Symptoms or Damaged Mucosal Barrier (*n*)	Patients with *K. kingae* Infection (*n*)	Percentage of Patients with Viral Symptoms (%)
Claesson, 1985 [13]	14	33	42.4
Yagupsky, 1993 [14]	14	25	56
Dubnov-Raz, 2020 [16]	200	322	62.1
Basmaci, 2015 [10]	10	17	58.9
El Houmami, 2018 [17]	8	9	88.9
Overall	246	406	60.6

**Table 2:** Description of virus or specific viral syndromes reported in published articles.

Virus/Viral Syndrome	Number of Patients Reported	First Author, Year
Human rhinovirus	12	Basmaci, 2015 [10]
	2	Basmaci, 2013 [19]
	1	Cardern, 1991 [20]
Coxsackievirus/hand, foot and mouth disease Or enterovirus	7	El Houmami, 2015 [21]
	5 *	El Houmami, 2015 [22]
	1 confirmed and 1 suspected	El Houmami, 2017 [23]
	1	Huard, 2016 [24]
	1	Sena, 2010 [25]
	2	Basmaci, 2015 [10]
Herpes simplex virus/stomatitis	4 (4 identified and 4 suspected)	Amir, 1998 [26]
	1	Serrera, 2021 [27]
Varicella zoster virus/chickenpox	1	Waghorn, 1997 [28]
	1	Amir, 1998 [26]
	1	Kampouroglou, 2016 [29]
Coronavirus OC43	4	Basmaci, 2015 [10]
Parainfluenzae	3	Basmaci, 2015 [10]
	1	Chosidow, 2019 [30]
Adenovirus	2	Basmaci, 2015 [10]
Epstein Barr virus	1	Hérissé, 2019 [31]
Influenza	1	Le Bourgeois, 2016 [32]
Total of confirmed cases	47	

* These 5 patients are probably duplicates from the study published by El Houmami et al. [21].

## Data Availability

Not applicable.
